# Desensitization of human breast progenitors by a transient exposure to pregnancy levels of estrogen

**DOI:** 10.1038/s41598-021-96785-8

**Published:** 2021-08-26

**Authors:** Lone Rønnov-Jessen, Jiyoung Kim, Nadine Goldhammer, Marie Christine Klitgaard, Martynas Smicius, Marc Baker Bechmann, René Villadsen, Ole William Petersen

**Affiliations:** 1 Section for Cell Biology and Physiology, Department of Biology, University of Copenhagen, 2100 Copenhagen Ø, Denmark; 2 Department of Cellular and Molecular Medicine, University of Copenhagen, 2200 Copenhagen N, Denmark; 3 Novo Nordisk Foundation Center for Stem Cell Biology, University of Copenhagen, 2200 Copenhagen N, Denmark

**Keywords:** Cell biology, Stem cells, Diseases, Endocrinology

## Abstract

Full term pregnancy at an early age is the only factor known to consistently protect against breast cancer. Because hormone receptor positive progenitors in the human breast relay endocrine signaling, we here sought to determine whether an experimental mimicry of the third trimester surge of hormones would change their susceptibility to growth stimulation. Hormone receptor positive, reduction mammoplasty-derived human breast epithelial progenitors were exposed to a short-term, pregnancy-level of estradiol, and their subsequent response to estradiol stimulation was analyzed. Exposure to pregnancy-level of estradiol results in subsequent lower sensitivity to estrogen-induced proliferation. Expression array and immunoblotting reveal upregulation of S100A7 and down-regulation of p27, both associated with parity and epithelial differentiation. Notably, we find that the epithelial differentiation is accompanied by upregulation of E-cadherin and down-regulation of vimentin as well as by diminished migration and more mature luminal epithelial differentiation in a mouse transplantation model. Our findings are in support of a de-sensitization mechanism for pregnancy-induced prevention against breast cancer.

## Introduction

The etiology of breast cancer and measures to reduce its incidence are still unresolved. In humans an apparent lifelong natural protection is obtained by full term pregnancy somewhat depending on the age at which pregnancy begins^1^. The greatest protection is obtained when the first full term birth occurs before the age of twenty, and parity continues to impact positively until the early thirties (reviewed in^2,3^). The protective effect only pertains to estrogen receptor-positive (ER^pos^) breast cancer, which is particularly interesting because of its relatively late onset in postmenopausal women^2^. That estrogens play a role in breast cancer prevention is suggested from studies in rodents where artificial imitation of the hormonal milieu of pregnancy confers the same protection as does pregnancy^4^. While the exact molecular mechanisms affording such protection are still far from understood (reviewed in^3^), it has been speculated that protection relates to either long-lasting cell autonomous alterations in the breast stem cell hierarchy or persistent changes in the systemic or microenvironmental hormonal milieu—or both^2^. Nevertheless, whether ER^pos^ cells participate in conveying this protection has remained an enigmatic question for decades. We and others have studied the role of estradiol (E2) on ER^pos^ normal human breast epithelial cells. In isolated primary reduction mammoplasty-derived cells E2 had a direct growth stimulatory effect on ER^pos^ progenitors^5^. Similar data were obtained in cells immortalized with human telomerase reverse transcriptase (hTERT) and short hairpin p16^6^. Given the bad luck hypothesis as an important determinant in cancer, cell division within a long-lived cellular compartment is to be considered a risk factor^7^. Indeed, E2 is listed as a carcinogen and the risk of breast cancer is slightly elevated immediately after birth-giving^2^.

Importantly, previous work did not address the role of pregnancy levels of E2 on ER^pos^ cells or whether such levels elicit any long-lasting changes. In theory, it could go both ways—either the cells become more transformed or less responsive to estrogens, the latter leading to a reduction in the life-long number of cell divisions, and in turn to a reduced risk of developing breast cancer.

Hitherto, knowledge of the influence of E2 on breast epithelial cells in general covers a wide range of hormone concentration. Exactly which level of E2 that should be applied to mimic pregnancy levels is, however, strongly indicated in a recent population based study where circumstances within the third trimester confers the protective effect of pregnancy^8^. This is where E2 levels in serum peak to a level of up to one hundred times higher than that of non-pregnant, reproductive-age-levels^9,10^. Also, insight into how normal breast ER^pos^ cells respond to pregnancy levels of hormone has so far been limited due to lack of appropriate models. We here employ a recently established normal breast luminal ER^pos^ epithelial cell line^6^ to explore the effect of a hormonal mimicry of pregnancy and describe its influence on estrogen sensitivity and cell differentiation.

## Results

**Figure 1:**
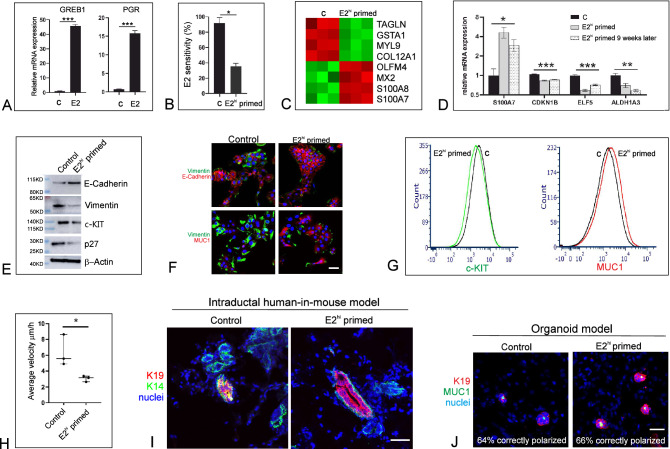
(**A**) iHBEC^ERpos^ are hormonally sensitive as shown by relative RT-qPCR of GREB1 and PGR upon E2 exposure (E2) versus control (C), fold expression mean + /− SEM, ***p < 0.001 by t-test. (**B**) Reduced relative increase in cell number in E2^hi^ primed cells versus control upon exposure to low E2, mean + /− SEM, *p < 0.05 by t-test. (**C**) Heat map of differentially expressed parity-related genes in E2^hi^ primed versus control created using R (version 3.6.3) and R studio (version 1.2.5033). Red and green indicate up- and down-regulation, respectively. (**D**) S100A7 up- and p27 (CDKN1B), ELF5 and ALDH1A3 down-regulated, respectively, in E2^hi^ primed cells (grey bar) versus control (black bar) and in E2^hi^ primed cells nine weeks later (dotted bar) versus its relative control, *p < 0.05, **p < 0.01 and ***p < 0.001 by ANOVA. (**E**) Up-regulation of E-cadherin and down-regulation of vimentin, c-KIT and p27 relative to β-actin by Western blotting in E2^hi^ primed versus control. The blots are grouped from different gels. (**F**) Confirmation by fluorescent staining of down-regulation of vimentin (green) and concurrent up-regulation of E-cadherin and MUC1 protein (red) upon E2^hi^ priming. (**G**) Reduction of c-KIT and increase of MUC1 expression by FACS analysis upon E2^hi^ priming. (**H**) Reduction of average velocity of E2^hi^ primed compared to control in scatter dot plot with median and interquartile range, *p < 0.05 by t-test. (**I**) In vivo, control cells form more K19^+^/K14^+^ profiles, while more mature ducts with K19^+^ luminal cells only (red) form from E2^hi^ primed cells as observed in cryosections of HIM transplanted glands. (**J**) E2^hi^ priming does not affect the ability of K19^+^ cells (red) to form acinus-like MUC1^+^ (green) structures. (**F,I,J**; bar = 50 µm).

E2^hi^ priming desensitizes ER^pos^ progenitors. For the sake of reproducibility we took advantage of a cloned recently established reduction mammoplasty-derived immortalized human breast epithelial ER^pos^ cell line (iHBEC^ERpos^)^6^, which expresses ER in 35% and PR in 9% of the cells and is hormonally responsive as revealed by induction of GREB1 and PGR in response to low E2 (Fig. 1A). The cells were exposed to a protocol with or without an up to two weeks transient pregnancy-level addition of E2 and progestin (P) (E2^hi^ priming) followed by a hormone-free interval to reestablish ER expression until the launch of a subsequent E2 sensitivity experiment with low E2. As seen in Fig. 1B the E2 sensitivity in terms of increase in cell number is significantly lower upon E2^hi^ priming (Fig. 1B). Since, in theory, the length of time periods of hormone exposure or deprivation may alter the proliferative response, the robustness of the response was confirmed in additional experiments with alternative periods of hormone exposure. Desensitization was not further augmented by additional rounds of E2^hi^ priming. High E2 alone resulted in similar desensitization, but there was no effect of P alone. This shows that E2 elicits a growth response in ER^pos^ progenitors, which is mitigated by prior E2^hi^ priming.

E2^hi^ priming reduces p27 expression and differentiates progenitors. To directly couple the apparent E2 desensitization with in vivo pregnancy related cellular profiling we performed a global gene expression analysis using RNA sequencing (RNA-Seq), which identified seven differentially expressed genes that were downregulated and 28 differentially expressed genes that were upregulated upon E2^hi^ priming (Supplementary Table 1). While acknowledging that the model employed here is much less complex than the physiological context in situ, we nevertheless found it interesting that several of the highly differentially expressed genes had previously been annotated to parity in humans by others (Fig. 1C)^11,12^. Specifically, S100A7 has been shown to be the second highest parity induced gene in uncultured luminal breast epithelial cells^11^. Since S100A7 is known to impact positively on human breast differentiation and cyclin-dependent kinase inhibitor 1B/p27^Kip1^ (p27)—a marker of breast progenitors negatively regulated and downstream of S100A7^11,13,14^—we focused on the dual expression of these genes along with the established progenitor markers ELF5 and ALDH1A3^15,16^. Significantly, both two- and nine weeks after E2^hi^ priming these genes remained up- and down-regulated, respectively (Fig. 1D). This is in strong favor of a stable E2^hi^ primed induction of maturation from a progenitor state of differentiation. To substantiate this we examined the response to E2^hi^ priming with classical epithelial transition markers along with p27 at the protein level. We found an increase of E-cadherin along with a decrease in vimentin, c-kit/CD117, and p27 (Fig. 1E and Supplementary Fig. 1), and regulation took place in both culture conditions tested, that is upon E2^hi^ priming in TGFβR2i without epidermal growth factor (EGF) or in TGFβR2i-1 with charcoal-stripped fetal calf serum (CCS). Immunocytochemistry and fluorescence activated cell sorting (FACS) analysis including the luminal differentiation marker sialomucin-1 (MUC1)^17^ confirmed a more differentiated phenotype upon E2 priming (Fig. 1F,G). Reduction of frequency of p27^+^ cells upon E2^hi^ priming was further confirmed in four sets of 6th passage finite lifespan CD117^high^ ER^pos^ epithelial cells (79.5% + /− 4.1 vs 52.4% + /− 10.9, p < 0.05 by t-test). The impact of E2^hi^ priming on epithelial functional differentiation was further reflected in a random motility assay where the E2^hi^ primed cells were found to be significantly less migratory (Fig. 1H). These results suggest that pregnancy levels of hormones favor the epithelial characteristics of luminal progenitors.

E2^hi^ primed progenitors maintain a normal luminal phenotype. It is well established that whereas human breast progenitors are double positive for keratin K19 and K14 (K19^+^/K14^+^), fully differentiated luminal cells express keratin K19 only^18^. Here we sought to recapitulate the in vivo differentiation state of the E2^hi^ primed cells by use of the intraductal human-in-mouse (HIM) transplantation model^19^. We found that more ducts derived from control cells showed few or no lumina and were keratin K19^+^/K14^+^ (12/21 structures) than from E2^hi^ primed cells (4/21 structures), which rather formed relatively more mature ducts with prominent luminal, correctly polarized and K19^+^ cells only (17/21 structures, Fig. 1I). Human origin of K19^+^ cells was ensured by use of a human-specific K19 antibody. Finally, to exclude that E2^hi^ priming transformed the cells we tested their performance in an organoid model, in which single epithelial cells are plated on human normal breast fibroblast feeders and recapitulate morphologically relevant normal-like three-dimensional structures^6^. Here, E2^hi^ primed cells did not differ significantly from control cells in their ability to form acinus-like structures with polarized expression of MUC1 (65.7 ± 2.3% MUC1-positive colonies from E2^hi^ primed versus 64.3  ±  1.5% from control cells, Fig. 1J). These findings suggest that pregnancy levels of E2 lead to maturation rather than transformation of luminal progenitors.

We conclude that a transient dose of pregnancy levels of E2 renders ER^pos^ cells less sensitive to subsequent induction of proliferation by E2 stimulation, and concurrently induces differentiation of progenitors into mature luminal cells.

## Discussion

We here demonstrate that a hormonal mimicry of pregnancy in terms of a transient exposure to pregnancy-level E2 renders ER^pos^ progenitors less sensitive to subsequent treatments with E2, and more importantly, this effect appears to persist for several generations. Since estrogen is a known driver of breast carcinogenesis, such reduction in estrogen sensitivity is likely to be relevant for cancer prevention, but has been difficult to test experimentally because of lack of models of normal-derived ER^pos^ cells. Previous studies in rodents have suggested that the oncoprotective effect of parity is due to a persistent decrease in mammary stem cells with repopulating activity^20^, thus implicating stem cells rather than progenitors. In line with the present findings, however, a recent study points to progenitors as mediators of the effect^21^. While use of stem cell markers confirmed a reduced growth response and repopulating activity in the parous gland, parity and additional rounds of pregnancy did not reduce stem cell numbers or function, and instead implicated cells outside the stem cell population^21^.

Estrogens and progesterones are major drivers of normal breast development but are also known to promote carcinogenesis^22^. We here show that rather than undergoing transformation, hormone exposed progenitors mature into highly differentiated luminal cells, and down-regulate p27 and upregulate S100A7—alterations associated with women´s reproductive history in vivo^11^. While the sequence of events and the interaction between the individual molecular players await further scrutiny, other studies lend support to the present findings. Thus, the regulation of p27^+^ hormone-responsive progenitors in human normal breast has been suggested as a marker of breast cancer risk^11^, and more specifically, deletion of *Cdkn1b* in rats leads to generation of a higher proportion of mature mammary luminal cells^23^, further implicating the pool size of hormone-responsive luminal progenitors in breast cancer risk. The role of S100A7 in normal breast also remains to be elucidated. However, that S100A7 expression may relate to a more differentiated state is suggested by its increased expression upon differentiation of normal keratinocytes (commented in^24^), and by the finding that induction of S100A7 in a normal-derived breast epithelial cell line results in a more differentiated luminal phenotype^13^.

Finally, we demonstrate that E2^hi^ primed cells undergo a mesenchymal-to-epithelial-like transition by up-regulating E-cadherin and down-modulating vimentin while simultaneously becoming less migratory suggesting a direct differentiating effect of pregnancy levels of E2 on ER^pos^ progenitors. In general, the reverse process, i. e. epithelial-mesenchymal transition, is considered a driving force of initiation, growth, invasion and metastasis of cancer^25,26^. Although the significance of the plasticity within the spectrum of EMT cell phenotypes in vivo has yet to be unraveled^25,26^, it is, nevertheless, tempting to speculate that a stabilization of the luminal epithelial phenotype as observed here upon exposure to pregnancy-level E2 would render the cells less susceptible to cancer development. Taken together, our results propose that E2^hi^ priming elicits a response in the pool of ER^pos^ progenitors, which offers a plausible explanation for the preventive effect of an early pregnancy on the risk of developing breast cancer later in life.

## Materials and methods

### Human cell culture

The immortalized human breast epithelial estrogen receptor-positive cell line, iHBEC^ERpos^, originally established from Ks20.8^pos^/CD166^high^/CD117^low^ cells isolated from normal breast and immortalized with hTERT and shp16^5^, was sorted as EpCAM^pos^/CD117^high^ in passage 35, cloned upon FACS as CD117^high^ in passage 42 and selected based on a high level of hormone receptor expression (ER in 35%, progesterone receptor (PR) in 9% of the cells) and its ability to form acinus-like structures on fibroblast feeders^6^, reminiscent of luminal cells´ morphological behavior in situ. The iHBEC^ERpos^ cell line is routinely maintained in TGFβR2i-1 in Primaria™ tissue culture flasks^6^. Primary CD117^high^ epithelial cells competent to express ER upon appropriate induction^5^ and intralobular fibroblasts^5,27^ were isolated from two normal breast biopsies and expanded as described^5,27^. The culture media TGFβR2i^5^, TGFβR2i-1^6^ and BBMYAB^5,27^ suppress TGFβ signaling, which is essential for long-term ER expression^5,27^. All methods were carried out in accordance with relevant guidelines and regulations. The use and storage of human material have been approved by the Regional Scientific Ethical Committees (Region Hovedstaden, H-2-2011-052) and the Danish Data Protection Agency (2011-41-6722), and by the donors by written informed consent.

### Growth experiments

Cells were exposed to either 17-β-estradiol (high E2 (E2^hi^), 125 nM, E2758, Sigma-Aldrich) and R5020 (P, 1 µM, Promegestone, R5020, Perkin Elmer), below referred to as E2^hi^ priming, or E2^hi^ alone or P alone, or vehicle (96% EtOH), in TGFβR2i-1^6^ with charcoal stripped fetal bovine serum (CCS, Gibco) for 11–13 days, deprived of hormones 10–23 days prior to passage to TGFβR2i without epidermal growth factor (EGF)^5^ with CCS supplemented with low E2 (1 nM) or vehicle for 19–22 days. TGFβR2i without EGF was employed to lower the baseline proliferation in cultures exposed to low estrogen, and CCS was included to avoid the effect of any endogenous estrogen in serum. For second round of E2^hi^ priming or extended culture, cultures were continued in TGFβR2i-1 with CCS. Cell quantification (NucleoCounter, NC-200) was performed on triplicate/quadruplicate cultures for each condition in three independent experiments. The robustness of the response was confirmed in four additional experiments with triplicate/quadruplicate cultures for each condition in which high hormone exposure was extended up to 21 days and/or the period of hormone depletion prior to split and exposure to low estrogen was reduced down to 8 days.

### RT-qPCR and RNA-Seq

Total RNA was extracted from cultures in triplicate using TRIzol Reagent (Thermo Fisher Scientific) and Direct-zol™ RNA MiniPrep Kits (Zymo Research). Reverse transcription was made using High-Capacity RNA-to cDNA™ kit (Applied Biosystems). For RT-qPCR, ^29^TagMan gene expression arrays for GREB1 (Hs00536409_m1), PGR (Hs01556702_m1), S100A7 (Hs00161488_m1), CDKN1B (Hs00153277_m1), ELF5 (Hs01063022_m1), ALDH1A3 (Hs00167476_m1), and reference genes GAPDH (Hs02758991_g1), PGK1 (Hs00943178_g1), TFRC (Hs00951083_m1), and HPRT1 (Hs99999909_m1) were used. Normalized mRNA expression was calculated using the 2^−ΔΔCT^ method and relative mRNA expression was measured as compared to control conditions.

To assess the responsiveness of iHBEC^ERpos^ to low E2 (1 nM) or vehicle for one day, cells were grown in TGFβR2i-1 with CCS for a total of 29 days prior to RNA extraction for RT-qPCR. Prior to RT-qPCR analysis upon extended culture, cells were cultured in TGFβR2i-1 with CCS for nine weeks and four passages after E2^hi^ priming and compared with cells analysed two weeks after E2^hi^ priming. For RNA-Seq, cells were primed with E2^hi^ or vehicle in TGFβR2i-1 with CCS for 11 days, depleted of hormones for 13 days before splitting into triplicate cultures, and grown for another 18 days before RNA extraction. RNA-Seq and bioinformatics analysis were performed by Beijing Genomics institute as described^28^. In brief, six RNA samples were sequenced using BGISEQ-500 giving in average 27.53 M reads per sample. The average-mapping ratio with reference genome was 93.81%, the average-mapping ratio with gene was 78.66% and 20,829 genes were detected after filtering. Clean reads to reference transcripts were mapped using Bowtie 2 method^29^ and gene expression levels were calculated with the RSEM method^30^. Differentially expressed genes between groups were identified using the DESeq2 method based on the negative binominal distribution^31^.

### Western blotting

Cells were exposed to E2^hi^ and P or vehicle in TGFβR2i without EGF for 10 days, depleted of hormones for 7 days, split and cultured in TGFβR2i without EGF and 2i for 4 days before extraction and blotting as described^5^ using antibodies against E-cadherin (1:1000, NCH-38, DAKO), vimentin (1:1000, V9, DAKO), c-KIT (1:1000, clone 47233, R&D Systems), p27 ^KIP1^ (1:1000, clone 57, BD Biosciences), and β-actin (1:5000, AC-15, Sigma). That markers were regulated irrespective of media condition was confirmed in an alternative set-up with E2^hi^ and P or vehicle exposure for 13 days in TGFβR2i-1 with CCS, depletion for 14 days, split and exposure to TGFβR2i-1 for 3 days prior to Western.

### Immunostaining

Cultured cells and tissue sections prepared as previously described^5,32^ stained for ER (SP1, ready to use), PR (1:50, PGR636), MUC1, (1:100, 115D8) and p27^KIP1^ (1:100, SX53G8, Abcam) by immunoperoxidase and for E-cadherin (1:10, HECD-1), vimentin (1:10, 3B4, Dako), MUC1 (1:10), K14 (1:50, LL002), K19 (1:50, human-specific, clone BA16) by fluorescence. Nuclei were counterstained with DAPI.

### Intrastain and FACS

Cells were exposed to E2^hi^ and P or vehicle in TGFβR2i without EGF for 9 or 10 days, deprived of hormones for 7 or 10 days prior to passaging into TGFβR2i without EGF and 2i for 4 days before Intrastain according to the manufacturer´s instruction (DakoCytomation). In brief, cells were trypsinized into single cells and incubated with fixative (IntraStain Reagent A) for 15 min, then washed in HEPES/BSA/EDTA buffer and permeabilized with IntraStrain Reagent B and stained using CD117-PE (1:20, 104D2) or MUC1 (1:50, 115D8) with AF488 IgG2b (1:500) as secondary antibody for 45 min. After washing twice in buffer and filtering through 20 µm filter cups (Filcons), cells were analysed in BD FACSAria Fusion, and data were visualized using FCS Express DeNovo Software, version 6 (https://denovosoftware.com/).

### Migration analysis

Cells were exposed to E2^hi^ and P or vehicle in TGFβR2i without EGF for 11 days, deprived of hormones or vehicle for 8, 9 and 10 days, respectively, subcultured at low density without 2i for 24 h to release cells from migratory restraint before individual cell tracking for 12 h of three sets of cultures. Migration was tracked manually using NIS Elements (Nikon) and velocity calculated using Chemotaxis and Migration Tool software (ibidi, http://rsb.info.nih.gov/ij/plugins/track/track.html).

### Organoid model

Cells were exposed to E2^hi^ and P or vehicle in TGFβR2i without EGF for 11 days, deprived of hormones or vehicle for 8 days prior to plating as single cells in three sets of co-culture with normal breast intralobular fibroblasts for two weeks in BBMYAB with SB431542 and RepSox to allow acinus-like polarized structures to form as described^6^. Epithelial structures were quantified upon peroxidase staining with MUC1^6^ at 10 × magnification using an ocular grid, and given as mean + /− SD of three times fifty MUC1^+^ colonies per culture in three independent experiments.

### Intraductal HIM transplantation

Cells were exposed to E2^hi^ and P or vehicle in TGFβR2i-1 with CCS for 14 days, depleted of hormones for 10 days prior to intraductal injection of single-cell suspensions into glands of a total of 17 NOD.Cg-Prkdc^scid^IL2rg^tm1Sug^/JicTac mice (Taconic) as described^19^ without surgically opening of the mouse. Glands were harvested after 4 months and snap-frozen in n-hexane prior to cryosectioning. A total of 42 human-derived structures, 21 from each condition, were identified and stained. Animal experiments are approved by the Danish National Animal Experiment Inspectorate (2017-15-0201-01315).

### Statistics

Statistical analyses and the graphic presentation of data by R (version 3.6.3), R studio (version 1.2.5033)^33^ and GraphPad Prism (Version 8) (https://www.graphpad.com/scientific-software/prism/), and estimated p values by t-test or one-way analysis of variance (ANOVA) were employed as indicated with significance at *p < 0.05, ** p < 0.01, ***p < 0.001, respectively.

## Supplementary Information

Supplementary Information.
