# Supplementary material for: Desensitization of human breast progenitors by a transient exposure to pregnancy levels of estrogen
Source: Sci Rep. 2021 Aug 26;11:17232. doi: 10.1038/s41598-021-96785-8 (PMC8390656; doi:10.1038/s41598-021-96785-8)
Supplement: Supplementary file 1 — Supplementary Information. [file 41598_2021_96785_MOESM1_ESM.pdf]

Supplementary information

Desensitization of human breast progenitors by a transient exposure to pregnancy levels of estrogen

Lone Rønnov-Jessen, Jiyoung Kim, Nadine Goldhammer, Marie Christine Klitgaard, Martynas Smicius, Marc Baker Bechmann, René Villadsen and Ole William Petersen

Supplementary Table 1. Differentially expressed genes between control and E2^{hi} primed cells identified by RNA-Seq

Symbol	GeneID	Length	Average expression in Control	Average expression in E2^{hi} primed	log2FoldChange (E2$^{hi\ primed}$/Control)	Padj	Pvalue
NFASC	23114	9714	2806.07	1564.47	-0.842878821	3.67E-25	5.56E-29
**GSTA1**	2938	1276	2625.84	1516.97	-0.791586566	2.96E-23	5.98E-27
**MYL9**	10398	1162	1911.40	1208.74	-0.661122640	3.12E-16	2.05E-19
**COL12A1**	1303	10437	3624.16	2297.61	-0.657514269	1.67E-20	5.88E-24
**TAGLN**	6876	1184	8737.38	5562.30	-0.651518084	5.65E-16	3.99E-19
GSTA2	2939	1320	216.98	139.33	-0.639047837	1.02E-06	2.74E-09
ADCYAP1	116	3211	764.44	504.44	-0.599714229	2.42E-09	3.42E-12
CEACAM6	4680	2631	481.93	723.79	0.586740284	1.82E-08	3.40E-11
IVL	3713	2165	17.89	27.09	0.598531802	2.45E-08	4.83E-11
IFIT1	3434	4411	4072.38	6170.34	0.599478181	1.77E-08	3.22E-11
WIF1	11197	2240	342.22	519.75	0.602884967	6.25E-08	1.32E-10
**MX2**	4600	2961	692.75	1058.91	0.612185429	6.38E-09	1.03E-11
HSH2D	84941	2369	467.85	721.24	0.624422280	4.61E-10	5.81E-13
XAF1	54739	3475	1298.50	2004.07	0.626089213	1.17E-09	1.59E-12
SPRR1B	6699	641	24.36	37.66	0.628672210	1.01E-08	1.68E-11
CMPK2	129607	2576	2066.27	3239.01	0.648522092	3.50E-19	1.41E-22
CSTA	1475	838	51.41	80.60	0.648682043	1.30E-06	3.62E-09
ZBP1	81030	1801	100.84	159.32	0.659782502	6.81E-07	1.68E-09
RSAD2	91543	3512	1962.80	3116.59	0.667059194	2.96E-15	2.24E-18
KRT6C	286887	2345	28.57	45.57	0.673570482	1.77E-08	3.21E-11
CEACAM5	1048	3600	55.98	89.47	0.676522517	4.15E-07	9.62E-10
PLAT	5327	3104	146.90	236.02	0.684088258	5.72E-08	1.18E-10
HLA-DRA	3122	1312	323.91	534.71	0.723172730	9.06E-10	1.19E-12
CYP4Z1	199974	1907	352.08	594.20	0.755053289	2.99E-12	3.01E-15
**OLFM4**	10562	2935	6487.09	11034.02	0.766314071	1.68E-21	4.25E-25
CRNN	49860	1913	25.12	44.15	0.813289156	1.41E-13	1.14E-16
HLA-DPA1	3113	1703	146.57	258.40	0.818022687	9.10E-12	9.64E-15
CFH	3075	3529	100.23	183.78	0.874600846	2.41E-12	2.19E-15
KLHDC7B	113730	3008	425.21	785.69	0.885802455	4.93E-17	2.98E-20
**S100A8**	6279	532	55.62	113.84	1.033325057	4.93E-17	2.91E-20
SERPINB3	6317	1793	40.89	83.98	1.038225769	3.95E-19	1.79E-22
KRT1	3848	2451	43.06	89.23	1.051343664	3.35E-18	1.69E-21
**S100A7**	6278	450	47.38	102.74	1.116632231	1.65E-20	4.98E-24
KRT6A	3853	2450	255.07	614.31	1.268055021	2.35E-37	2.37E-41
CXCL13	10563	1219	1780.74	5871.38	1.721221231	4.70E-87	2.37E-91

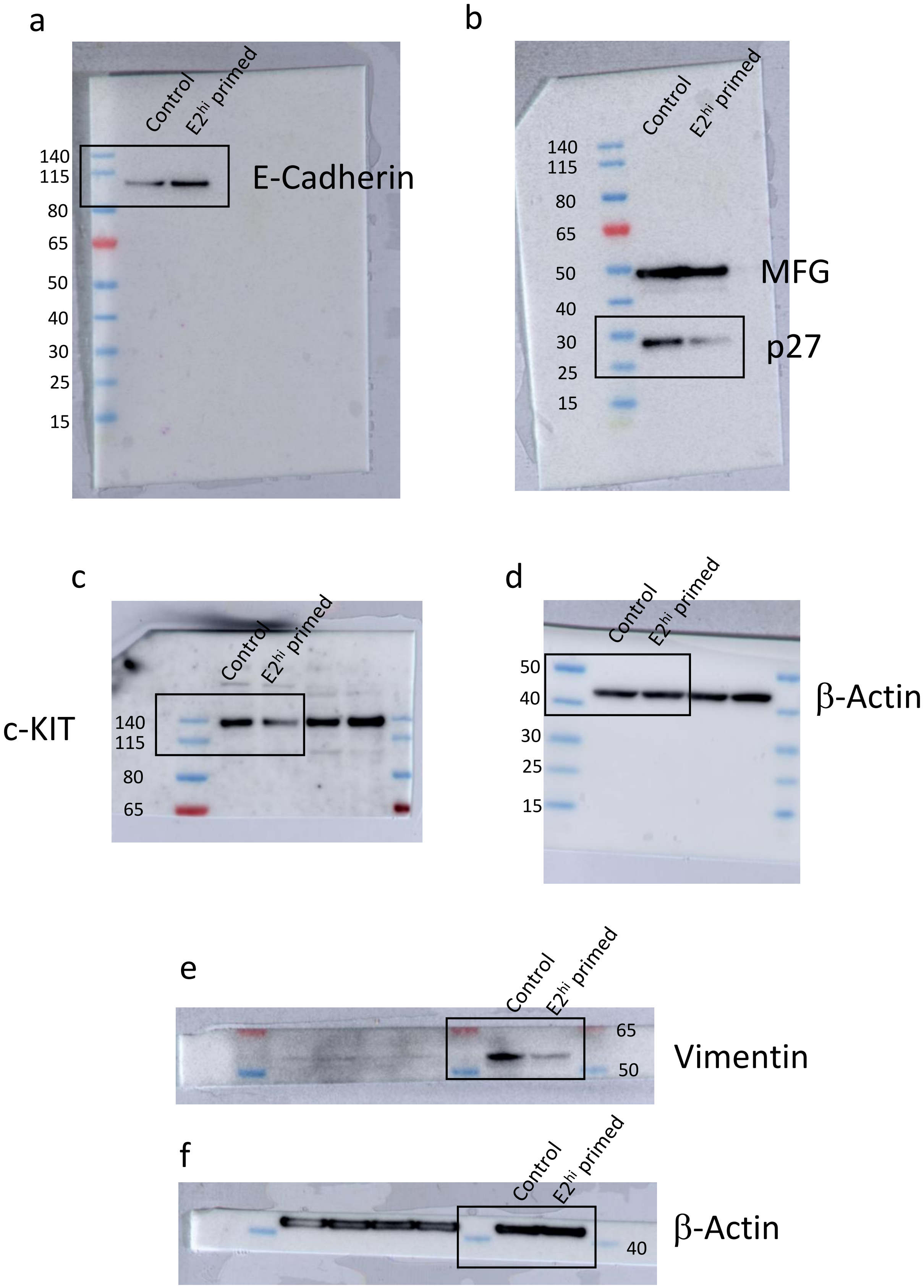

Supplementary Figure 1. Collection of blots grouped in Fig. 1E with framing of relevant parts. The amount of protein loaded in each case is 30 μg from Control and from $E2^{hi}$ primed cells, respectively, and molecular weight markers were included as indicated. Blots were incubated with antibodies recognizing E-cadherin (a), p27 (b), c-KIT (c), vimentin (e) and β-actin (d, f) as described in Materials and Methods. The p27 blot was incubated with an antibody not included in the study, anti-Milk Fat Globule monoclonal antibody (MFG, clone EDM45, Biomarker), prior to incubation with the antibody recognizing p27. β-actin was included as loading control, and membranes were cut prior to incubation with β-actin antibody, shown here for corresponding to c-KIT (d) and vimentin (f). The difference in intensity between d and f is due to difference in exposure time.
